# Supplementary material for: Structural and Functional Versatility of the Amyloidogenic Non‐Amidated Variant of the Antimicrobial Peptide Citropin 1.3
Source: Adv Sci (Weinh). 2025 Sep 28;12(46):e03997. doi: 10.1002/advs.202503997 (PMC12697819; doi:10.1002/advs.202503997)
Supplement: Supplementary file 1 — Supporting Information [file ADVS-12-e03997-s001.pdf]

Supplemental Information

Structural and Functional Versatility of the Amyloidogenic Non-Amidated Variant of the Antimicrobial Peptide Citropin 1.3

Fabio Strati, Mariana Pigozzi Cali, Yehudi Bloch, Siavash Mostafavi, Jim Monistrol, Aleksandr Golubev, Bader Rayan, Emil Gustavsson, Meytal Landau*

The Supplemental Information contains:
Figures S1-S19

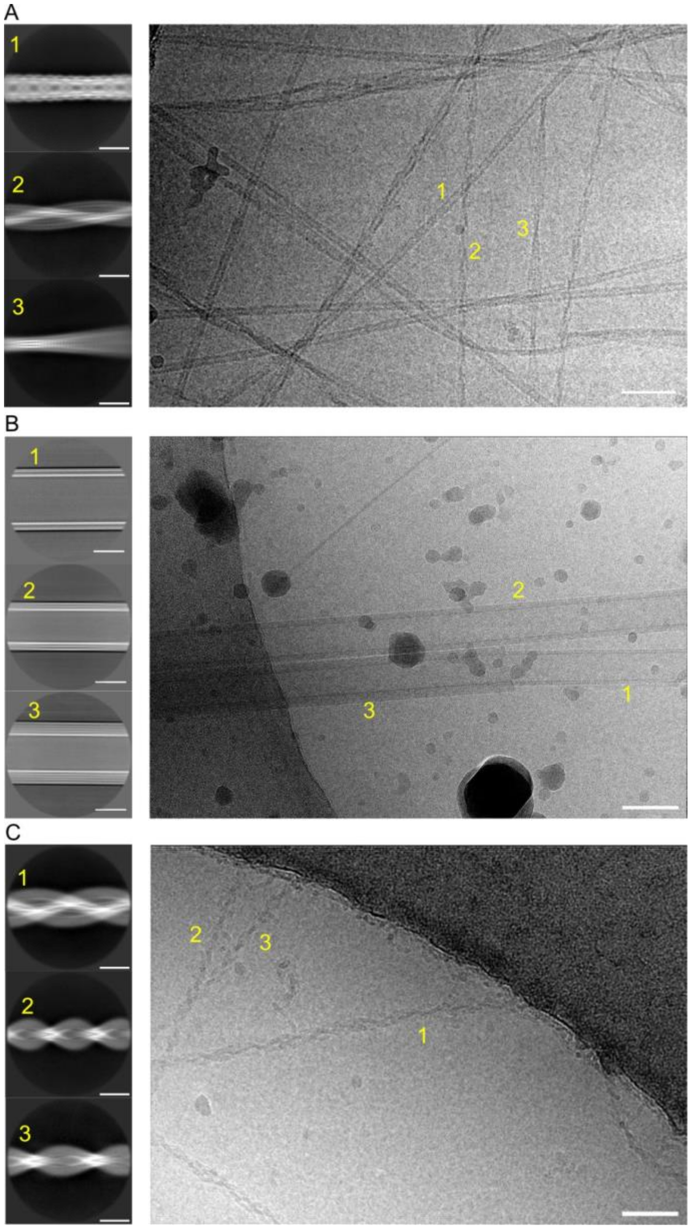

Figure S1. Cryo-EM representative micrographs and 2D class averages of citropin 1.3 datasets from different incubation conditions. **A)** 1 mM citropin 1.3 in 50 mM NaCl aqueous solution at pH 5, **B)** 1 mM citropin 1.3 in 10 mM PBS at pH 7.4 and **C)** 0.5 mM citropin 1.3 in 10 mM PBS in presence of 1.5 mM of DOPE:DOPG (2:1 molar ratio) liposomes. The different classes are numbered based on their relative particle count in each dataset. Scale bars, 500 Å for micrographs, 100 Å for 2D class averages in A) and C), 200 Å for 2D class averages in B).

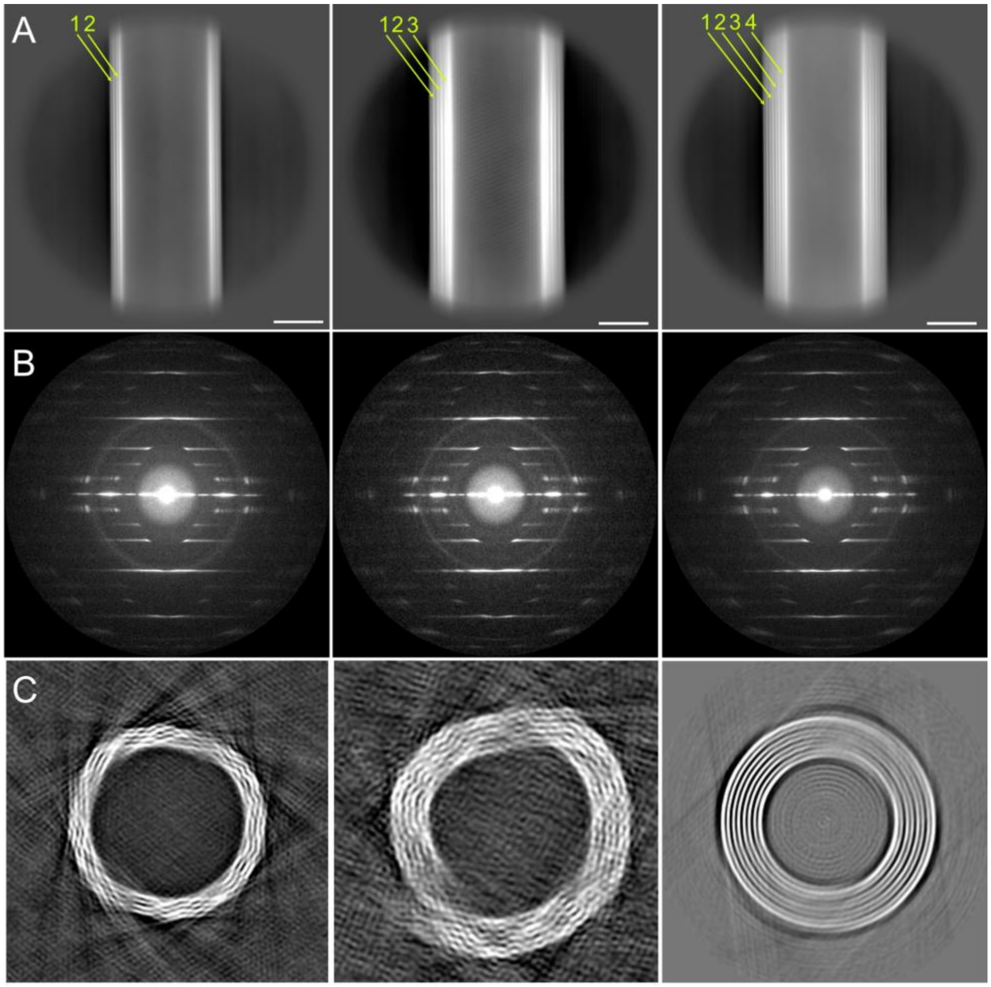

Figure S2. Visualization of citropin 1.3 nanotubes formed in PBS at pH 7.4. A) Representative cryo-EM 2D classes of obtained nanotubes with indication of number of layers for each class, scale bars are 200 Å. **B)** Power spectra corresponding to each 2D class. **C)** Cross-section of low-resolution 3D volumes obtained for each nanotube class.

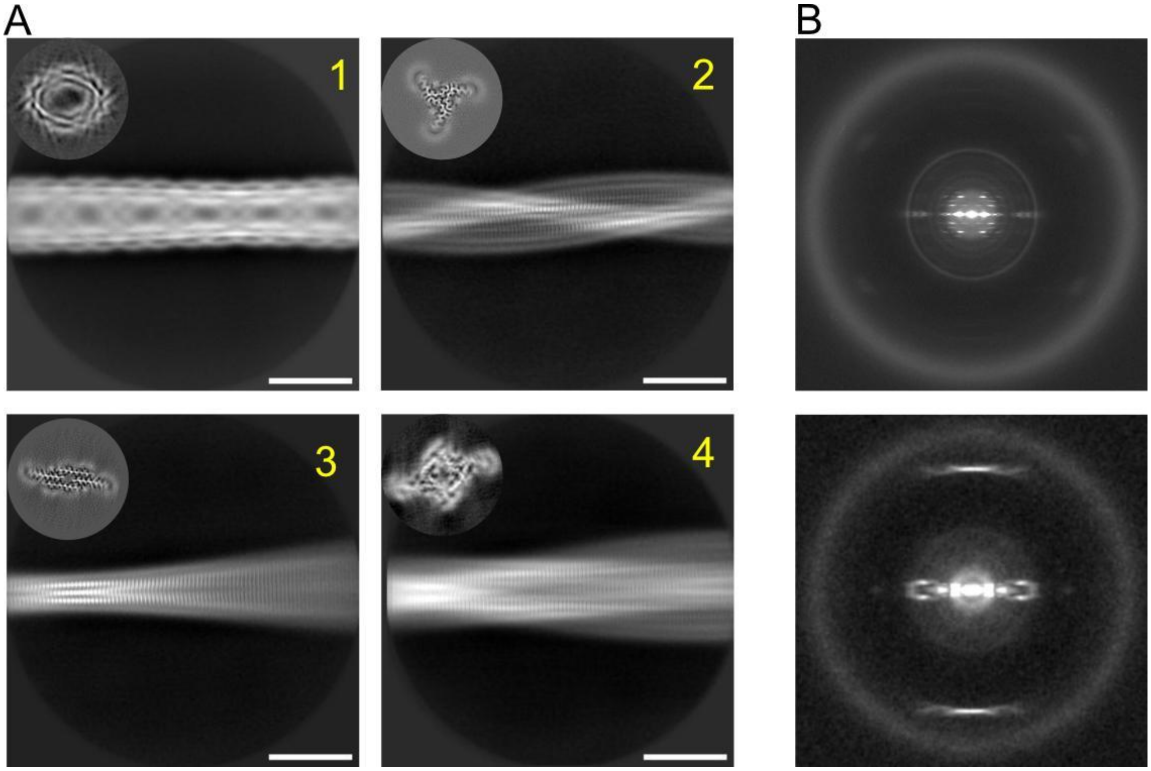

Figure S3. Two dimensional details of citropin 1.3 fibrils formed at pH 5. A) Cryo-EM 2D class averages of different polymorphs formed in 50 mM NaCl at pH 5, numbered 1 to 4, with 50.3%,16.2% 13.9%, 7.6% relative particle count per class on total percentage, respectively. The cross-sections of the relative class maps are shown as insets at the top right. Scale bars are of 50 Å. **B)** Obtained power spectra from Pol I (top) and representative amyloid class from Pol III (bottom).

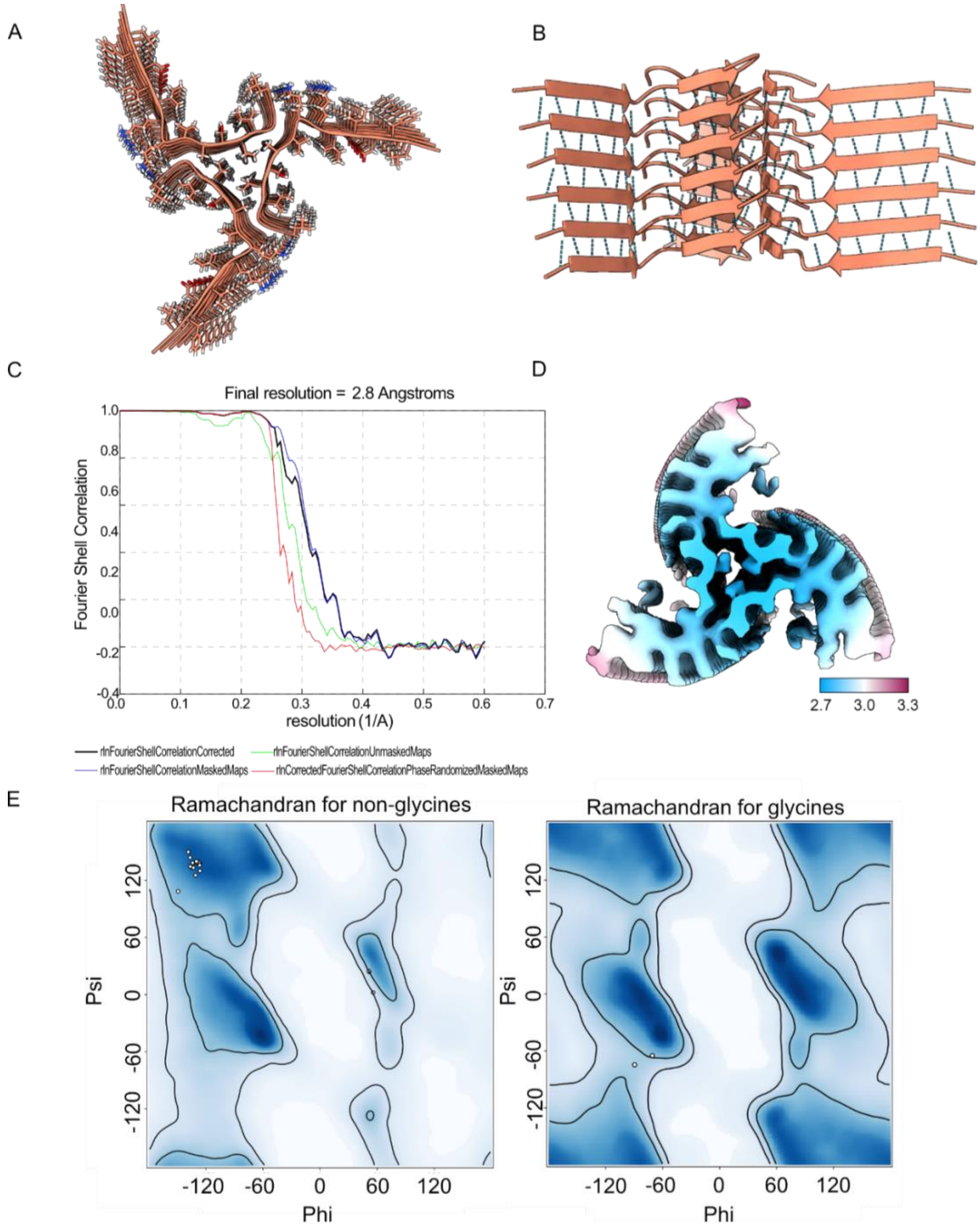

Figure S4. **Citropin 1.3 polymorph II cryo-EM map and model overviews. A)** A top view of the Pol II model with side chains shown as sticks. **B)** Side view with visualization of backbone hydrogen bonds. **C)** Gold Standard Fourier Shell Correlation (GSFC) curve reported from Relion of Pol II-L map and **D)** local resolution visualization of map cross-section. **E)** Ramachandran plots as reported from software Phenix for non-glycine and glycine residues. Most residues reside in

the β-sheet region, except from Lys7 and Lys8, which display angles close to a left-handed α-helix.

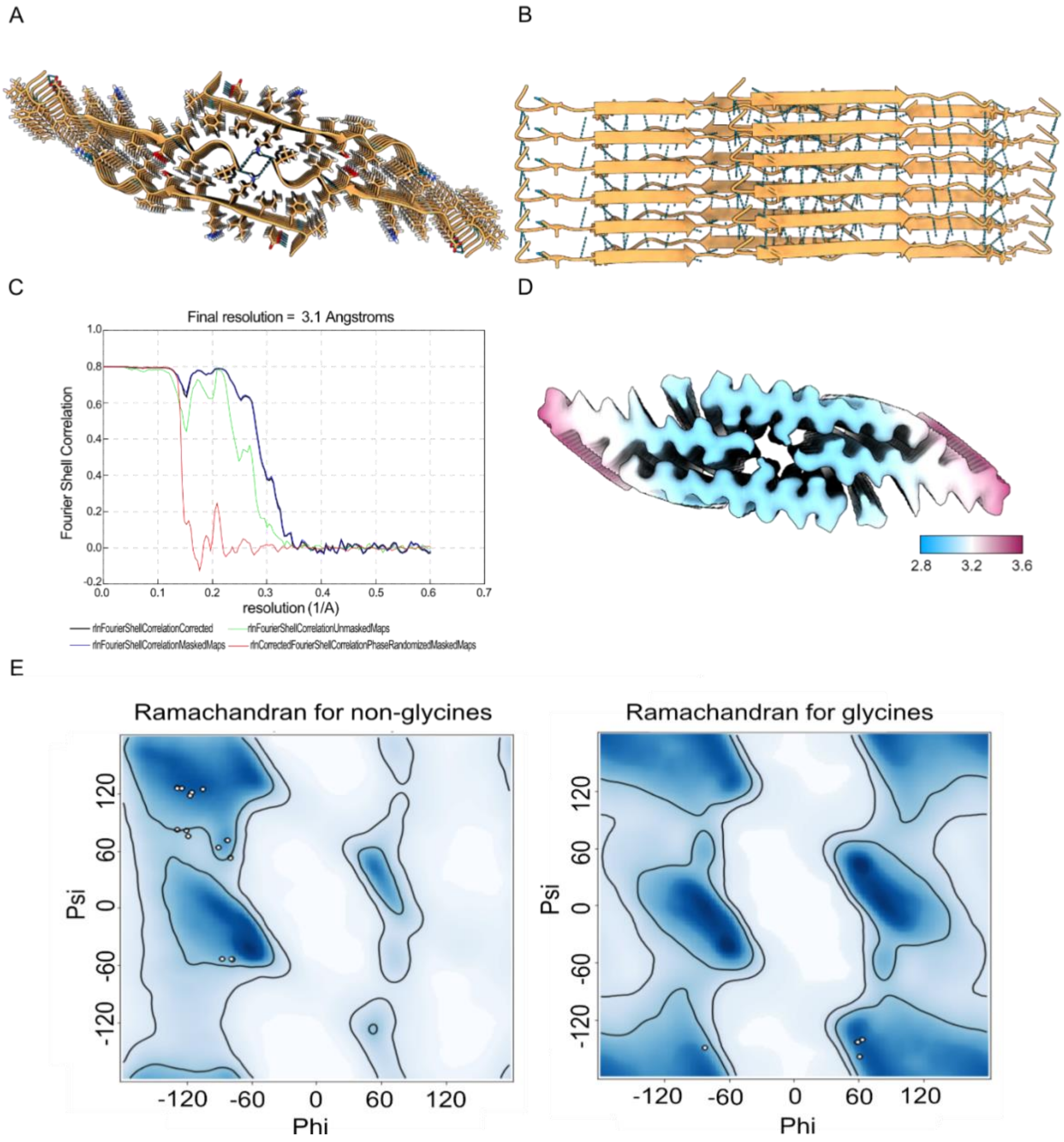

Figure S5. **Citropin 1.3 polymorph III cryo-EM map and model overviews. A)** A top view of the Pol III model with side chains shown as sticks. **B)** Side view with visualization of backbone hydrogen bonds. **C)** GSFC curve reported from Relion of Pol III-L map and **D)** local resolution visualization of map cross-section. **E)** Ramachandran plots as reported from software Phenix for non-glycine and glycine residues.

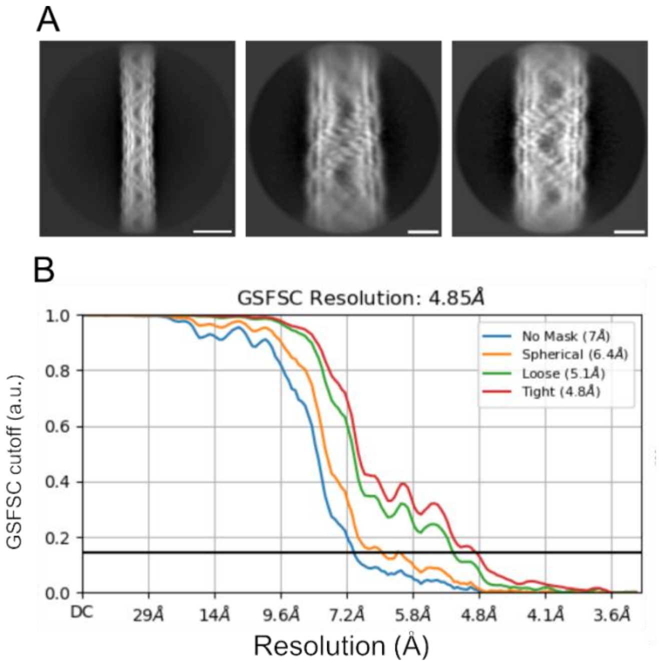

Figure S6. Citropin 1.3 polymorph I 2D classes and 3D refinements. A) Cryo-EM 2D classes of Pol I with 680 (left, scale bar of 100 Å) and 300 (middle and right, scale bar of 50 Å) pixels box size. **B)** Gold Standard Fourier Shell Correlation (GSFSC) curves with resolution reported by the CryoSPARC software at a cut-off at 0.143.

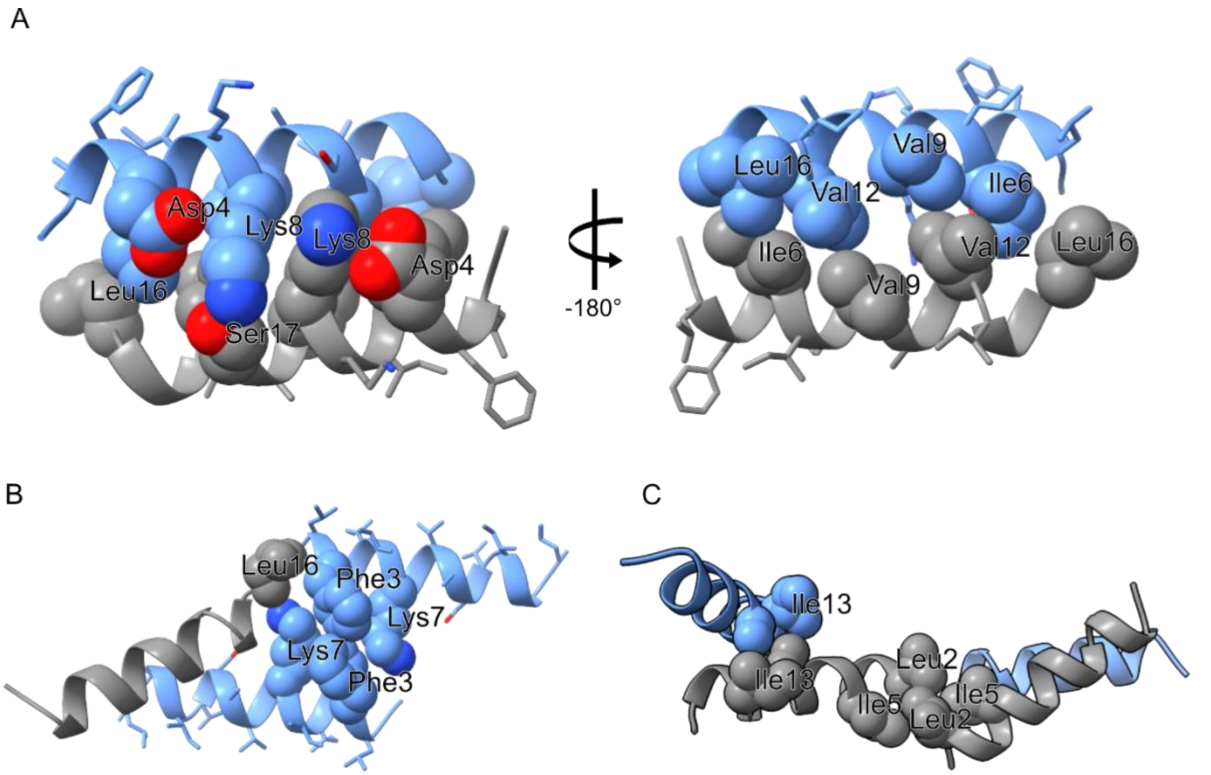

Figure S7. Inter-α-helical packings in the crystal structure of citropin 1.3. This figure highlights a subsection of the α-helical pairs in the citropin 1.3 crystal structure. Chains A and B are depicted in grey and light blue, respectively. **A)** The panel presents the interactions between the α-helices of chains A and B, shown in two orientations rotated approximately 180°. The helices exhibit tight packing mediated by hydrophobic interactions involving Ile5, Val9, Val12, and Leu16. Additionally, a hydrogen bond is observed between Lys8 on chain B and Ser11 on chain A. Each chain also features an internal electrostatic interaction between Lys8 and Asp4. **B)** The panel displays further stabilizing interactions between adjacent pairs, including tight packing between Phe3 and Lys7 of neighboring chain Bs, as well as interactions with Leu16 from chain A. **C)** The panel displays further stabilizing interactions between adjacent pairs, including tight packing between Leu2 and Ile5 of neighboring chain As, and between Ala10 and Ile13 of neighboring chain A and B. These interactions collectively contribute to the stability and organization of the α-helical assembly.

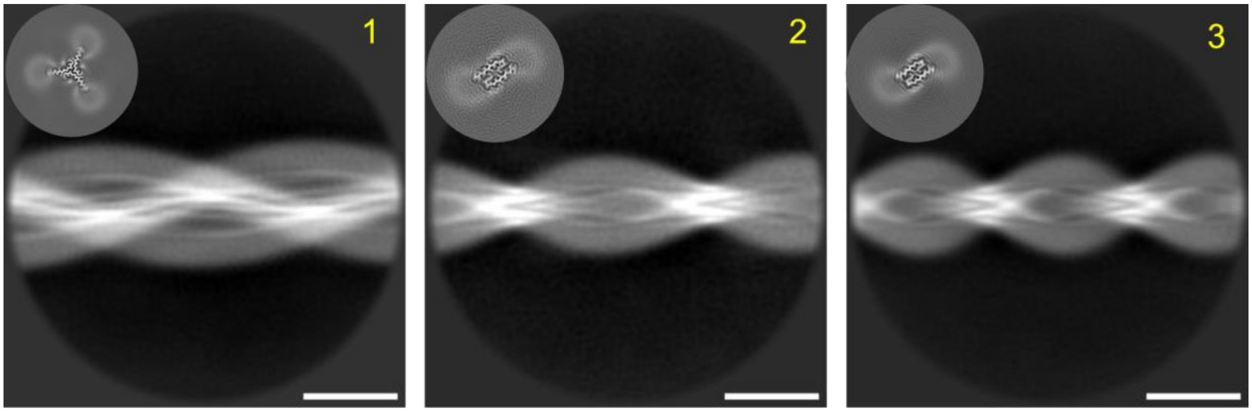

Figure S8. **Two-dimensional details of citropin 1.3 amyloids formed with liposomes**. Cryo-EM 2D class averages of different polymorphs obtained in PBS pH 7.4 in the presence of negatively charged liposomes in a 1:5 molar ratio (peptide:liposome). The polymorphs are numbered 1 to 3 representing 24.6%, 24.0%, 12.1% relative particle count per class on total percentage. The insets show the cross-sections of relative class maps. Scale bar 100 Å.

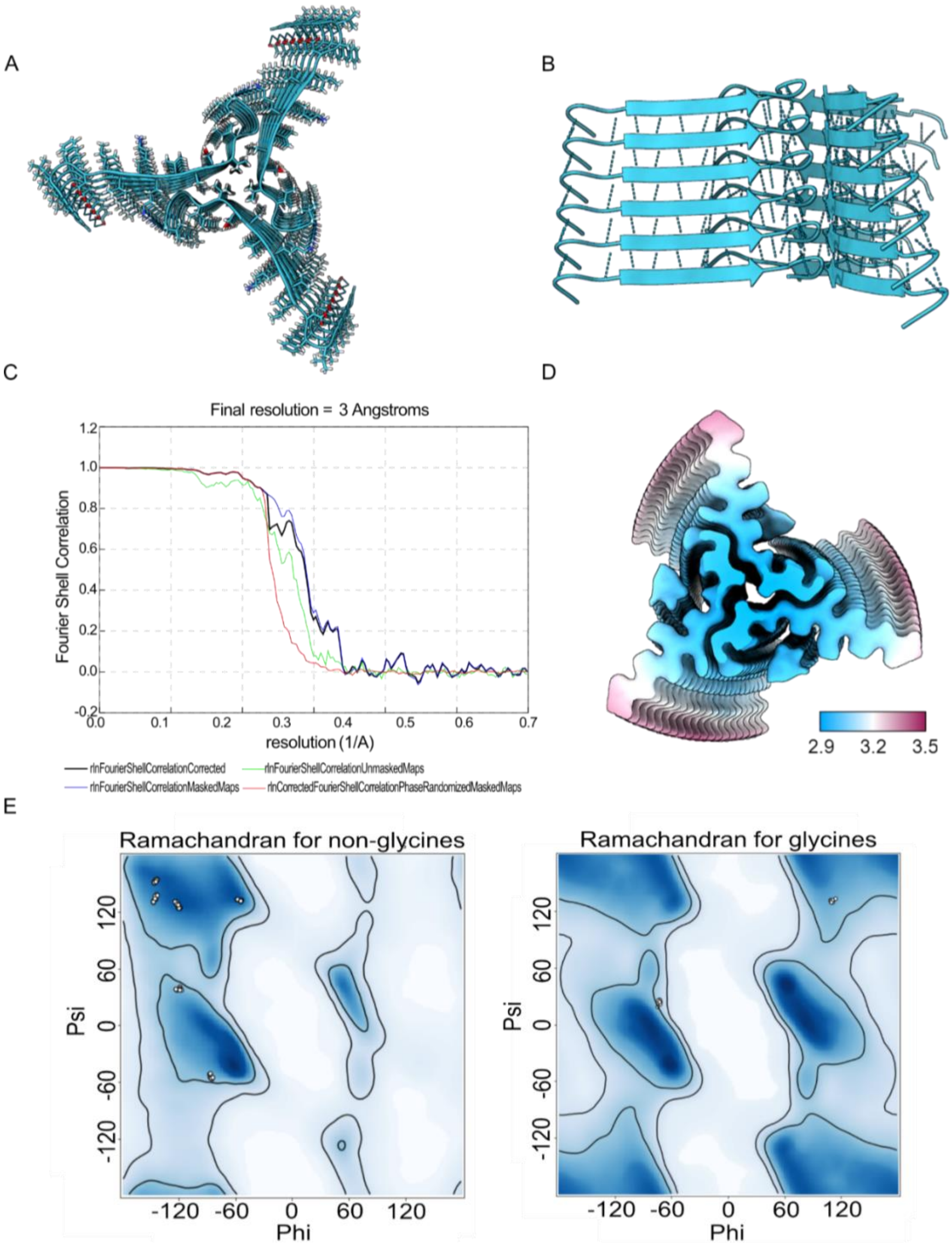

Figure S9. **Citropin 1.3 polymorph I-L cryo-EM map and model overviews. A)** A top view of the Pol I-L model with side chains shown as sticks. **B)** Side view with visualization of backbone hydrogen bonds. **C)** GSFC curve reported from Relion of Pol II-L map. **D)** Local resolution visualization of map cross-section. **E)** Ramachandran plots as reported from software Phenix for non-glycine and glycine residues.

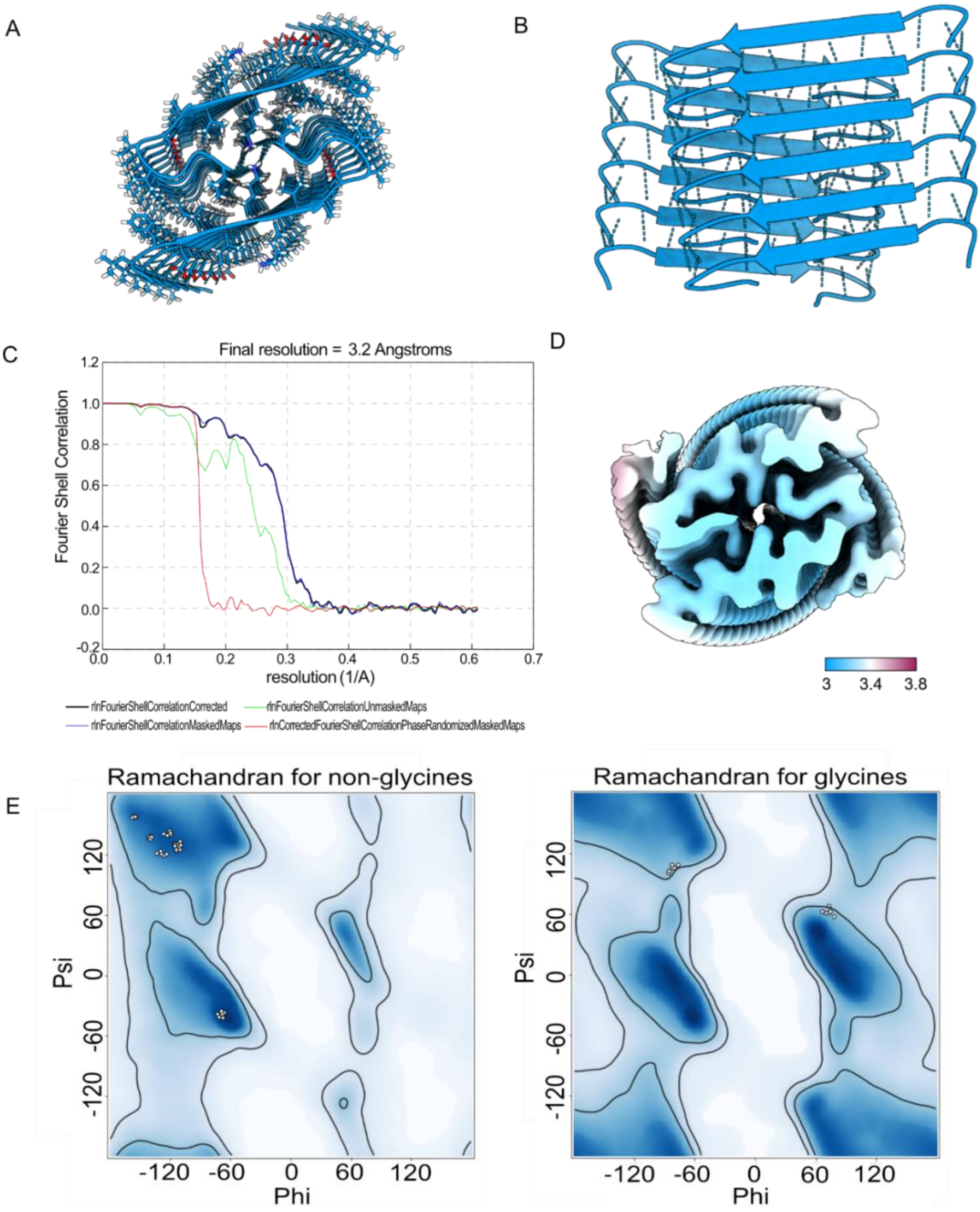

Figure S10. **Citropin 1.3 polymorph II-L cryo-EM map and model overviews. A)** A top view of the Pol II-L model with side chains shown as sticks. **B)** Side view with visualization of backbone hydrogen bonds. **C)** GSFC curve reported from Relion of Pol II-L map. **D)** Local resolution visualization of map cross-section. **E)** Ramachandran plots as reported from software Phenix for non-glycine and glycine residues.

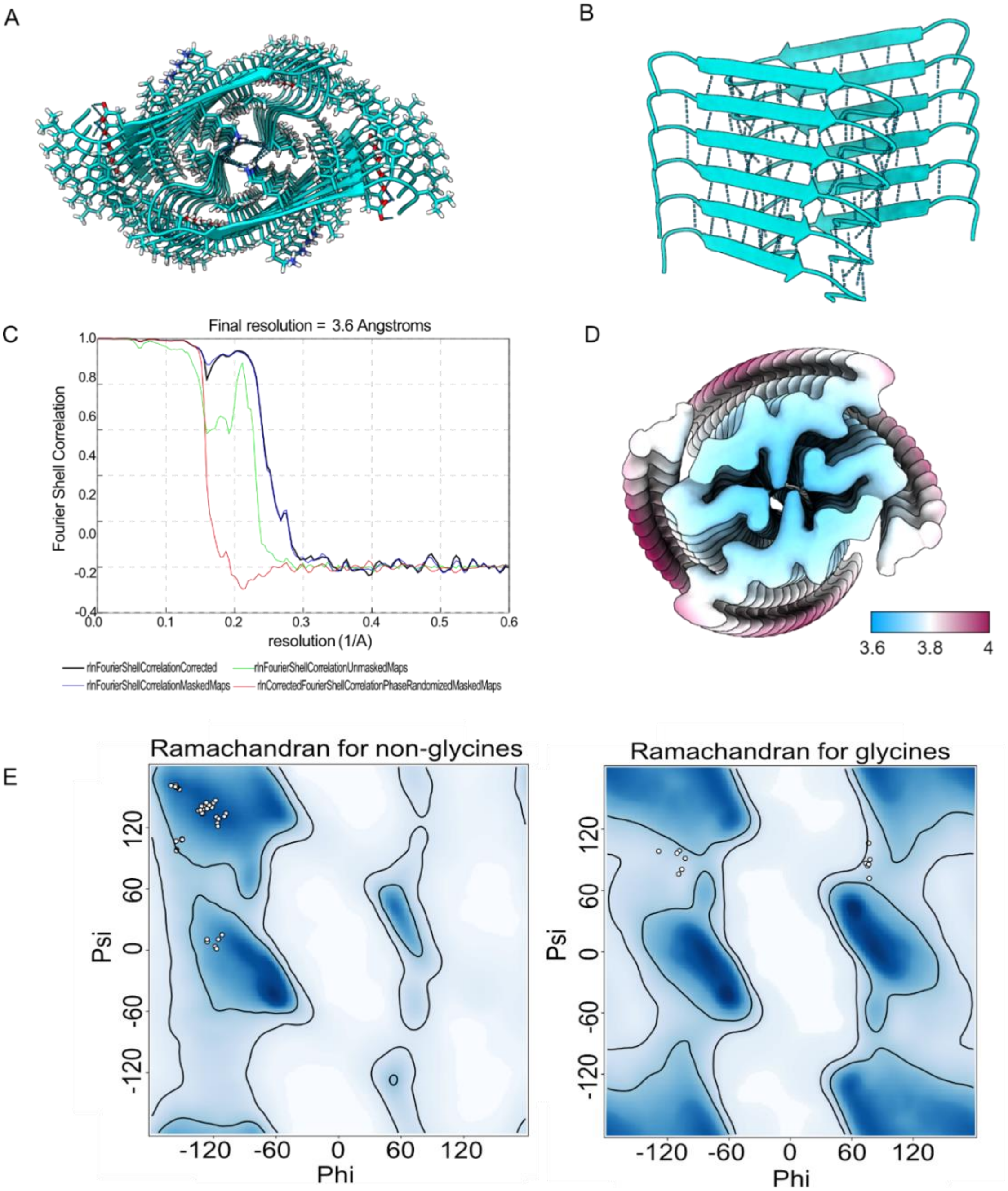

Figure S11. Citropin 1.3 polymorph III-L cryo-EM map and model overviews. A) A top view of the Pol III-L model with side chains shown as sticks. **B)** Side view with visualization of backbone hydrogen bonds. **C)** GSFC curve reported from Relion of Pol III-L map. **D)** Local resolution visualization of map cross-section. **E)** Ramachandran plots as reported from software Phenix for non-glycine and glycine residues.

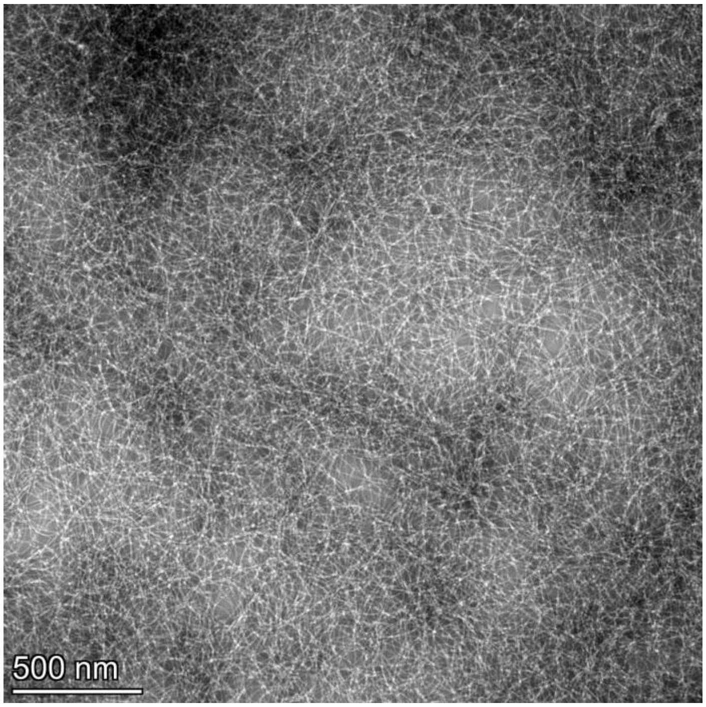

Figure S12. Negative stain TEM image of FITC-citropin 1.3. Negative stain TEM image of citropin 1.3 labeled with FITC at the N-terminal end, incubated for 24 hours at 0.5 mM in water. Scale bar 500 nm.

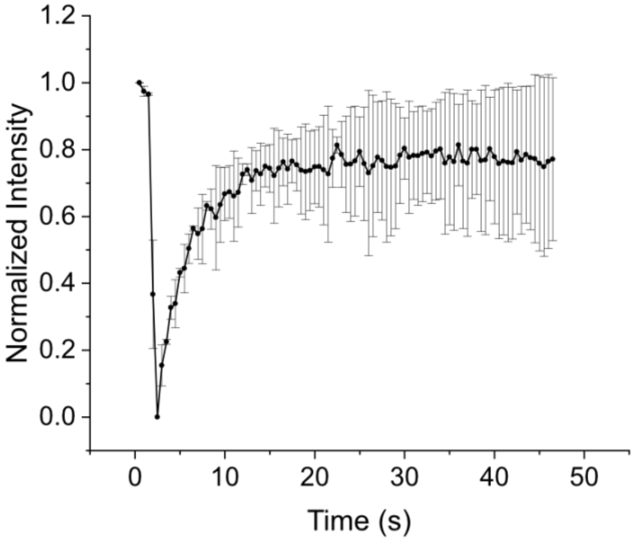

Figure S13. Fluorescence recovery after photobleaching of droplets. FRAP results after the interaction between DOPE:DOPG GUVs and citropin 1.3, as visualized in Figure 5B. The graph presents an average and error bars based on five measurements from two independent experiments.

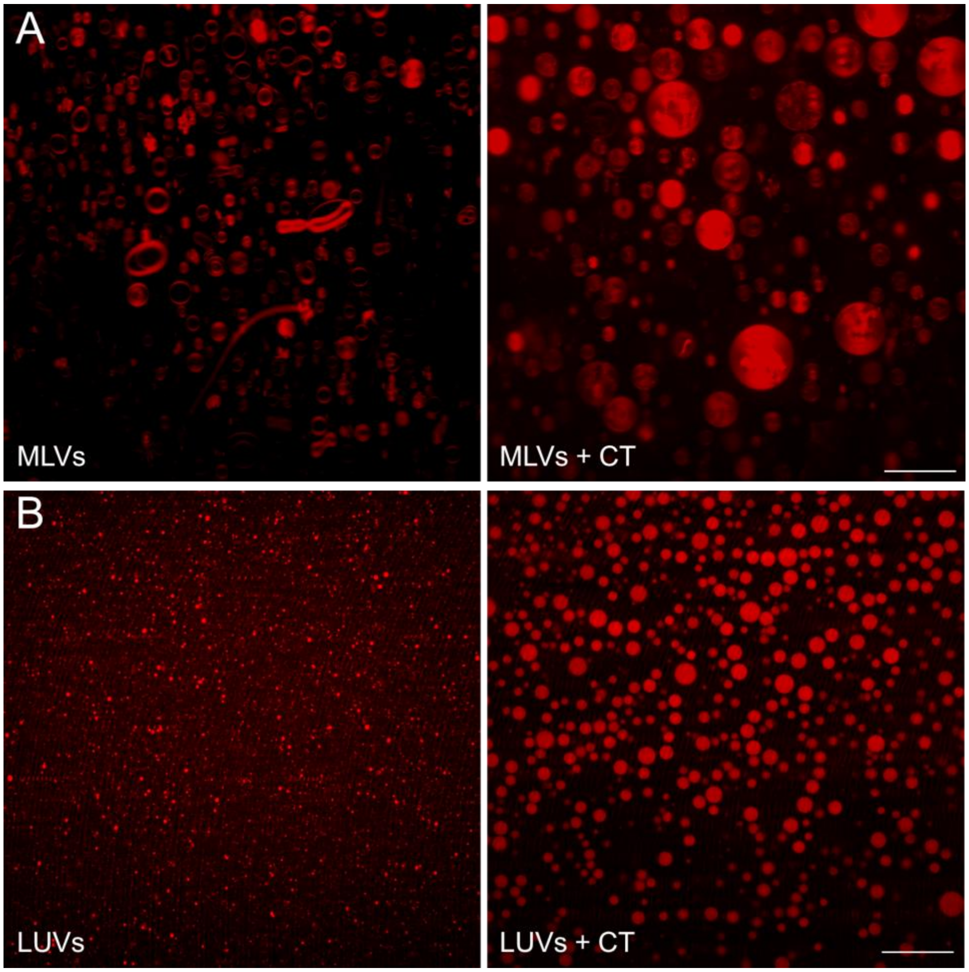

Figure S14. The effect of citropin 1.3 on liposomes composed of multilamellar and large unilamellar vesicles. Fluorescence microscopy visualization of: **A)** multilamellar vesicles labelled with 18:1 Lissamine Rhodamine-labelled PE before (left) and after (right) the addition of citropin 1.3 (CT). **B)** Large unilamellar vesicles before (left) and after (right) the addition of unlabeled citropin 1.3 (CT). Both panels show the formation of larger vesicles induced by peptide addition. Scale bar 20 μm.

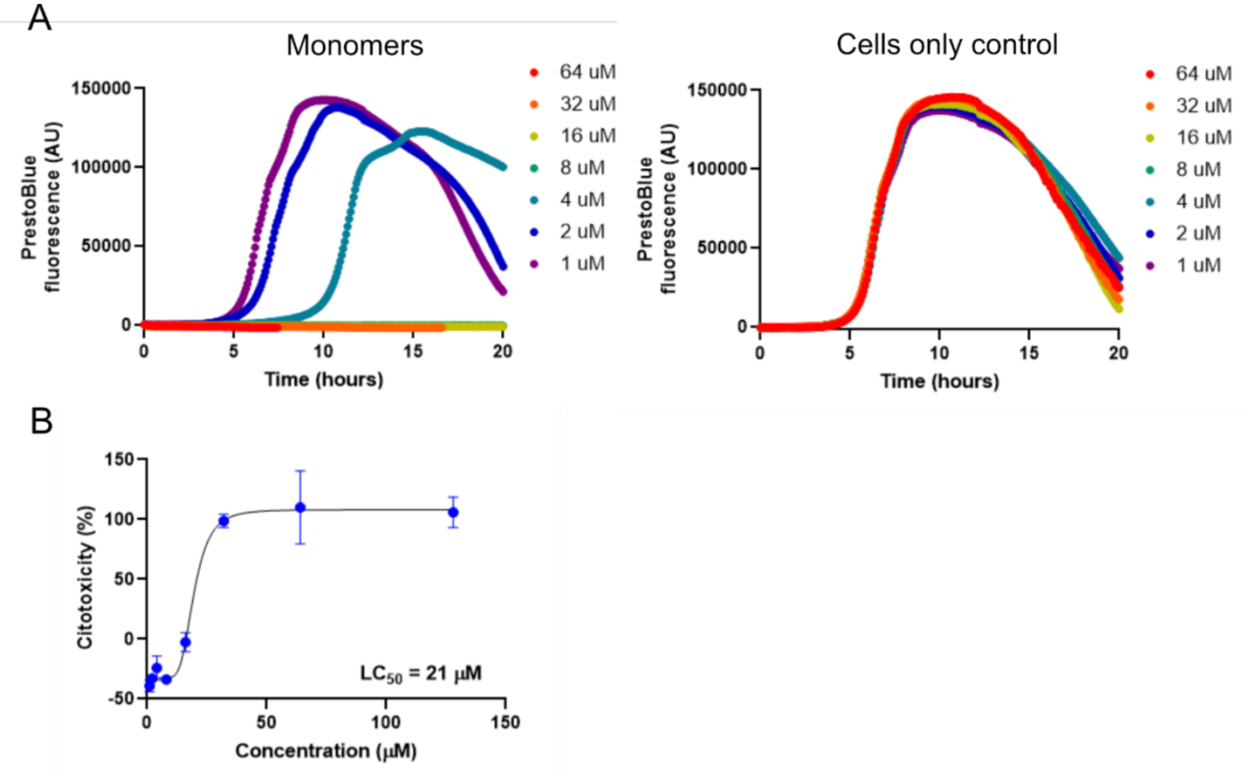

Figure S15. MIC and LC_{50} values for citropin 1.3. A) The left panel illustrates the growth of *Bacillus subtilis* in the presence of varying concentrations of citropin 1.3 (1 to 64 µM, as indicated by the color codes). The minimum inhibitory concentration (MIC) was determined to be 8 µM, which was sufficient to completely inhibit bacterial growth. The right panel depicts control experiments, where *Bacillus subtilis* growth was measured with the addition of a buffer at equivalent volumes corresponding to the peptide concentrations (color-coded to match the respective peptide volumes). **B)** The cytotoxicity assay for citropin 1.3 against A-549 cells. The lethal concentration at which 50% of the cells are killed (LC_{50}) was determined to be 21 µM.

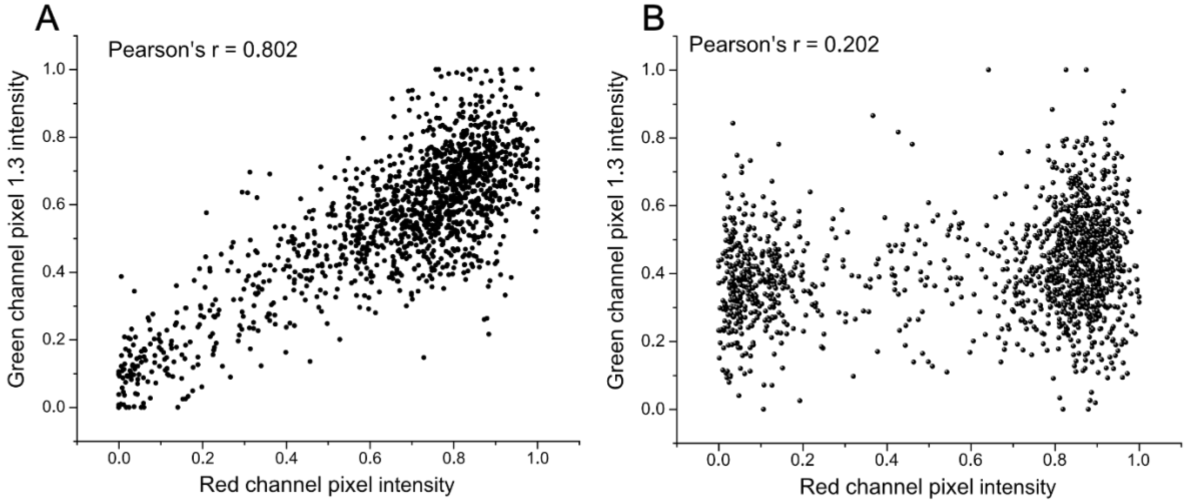

Figure S16. Scatter plot analysis of normalized pixel intensities from the red channel (propidium iodide) and green channel (FITC-labeled citropin 1.3) within the nucleolus of A-549 cells. A) Scatter plot showing pixel intensity correlation between propidium iodide (Cy5, red channel/633 nm) and 21 µM citropin 1.3 (1% FITC-labeled, 99% unlabeled; green channel/ GFP-475 nm) based on the fluorescence microscopy experiment shown in Figure 6A. **B**) Control condition in the absence of citropin 1.3, where Triton X-100 was used to induce cell death (Figure 6C). As expected, signal appears only in the red channel due to membrane permeabilization, resulting in low correlation. The Pearson's correlation coefficient (r) is shown for each condition and provides a quantitative measure of co-localization between red and green channel signals.

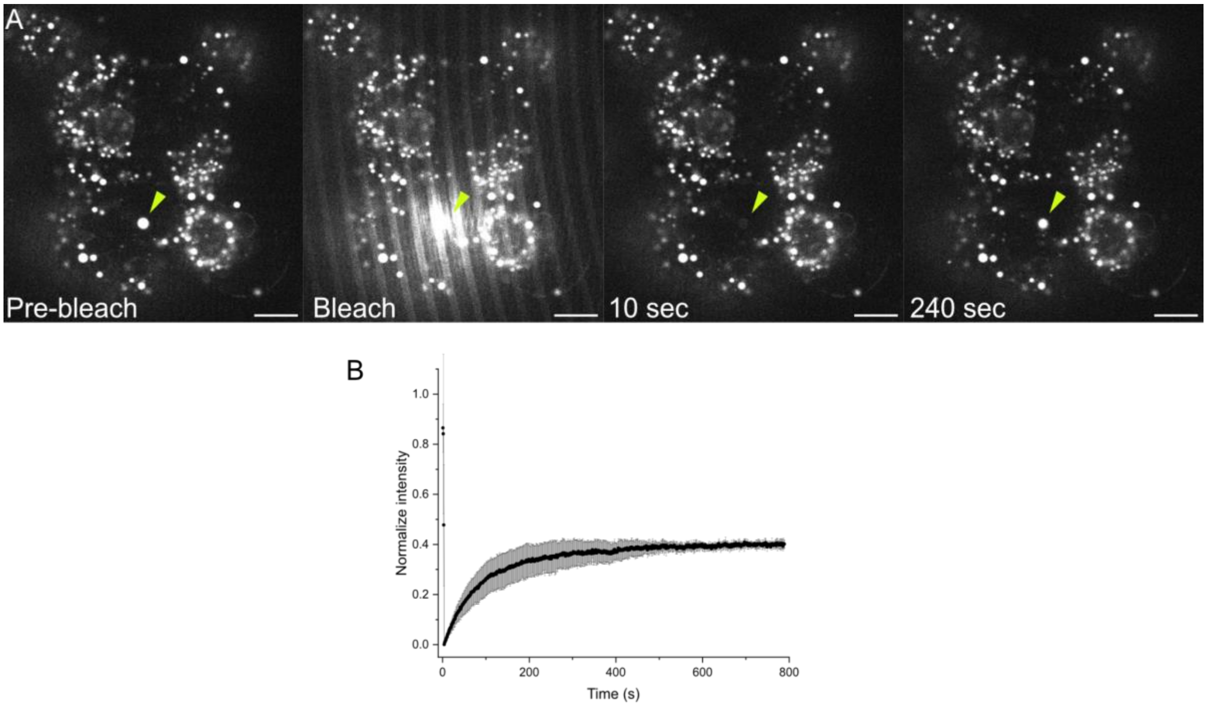

Figure S17. Fluorescence recovery after photobleaching (FRAP) of droplets formed by citropin 1.3 following A-549 cell lysis. **A)** FRAP experiment performed on a single droplet enriched with citropin 1.3 (marked by a yellow triangle) demonstrates fluorescence intensity recovery over time after bleaching. **B)** Graphical representation of the FRAP results, showing the average fluorescence recovery curve from four independent measurements. Scale bar 10 μm.

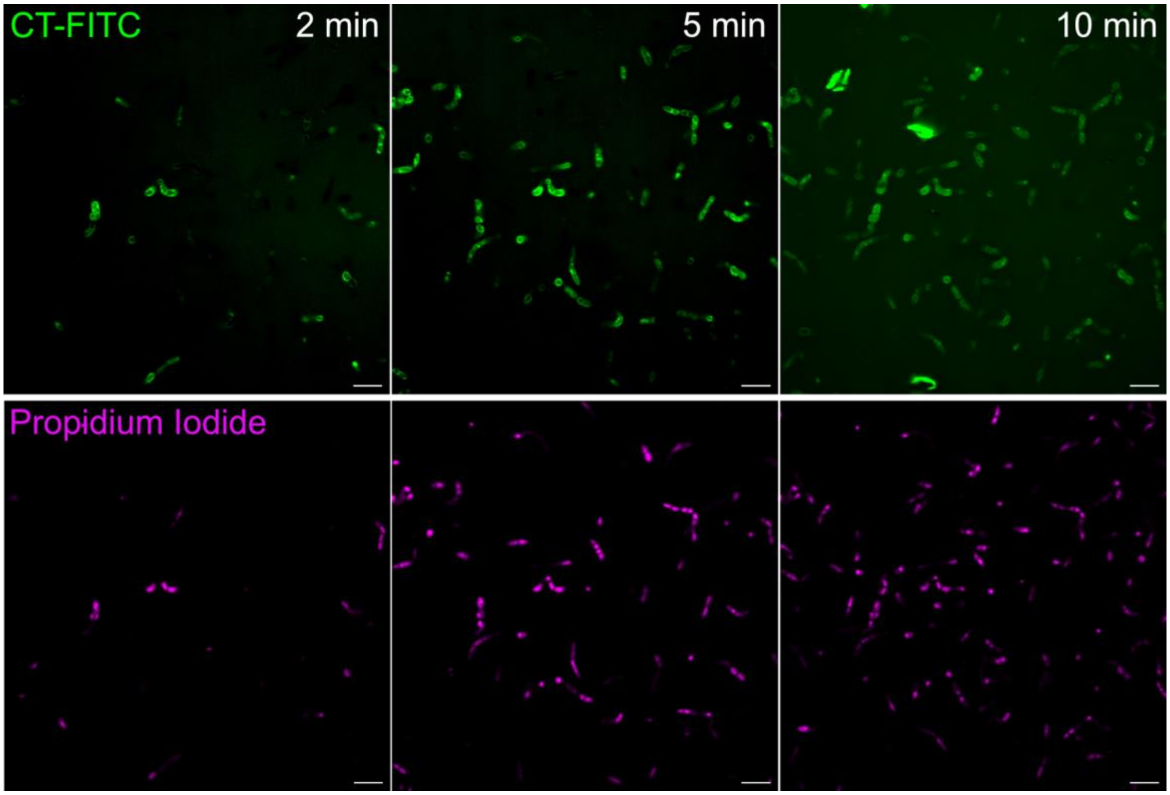

Figure S18. **Citropin 1.3 interactions with *Bacillus subtilis*.** Fluorescence microscopy visualization of FITC-labeled citropin 1.3 (CT-FITC) interacting with the bacterial cells, causing cell death as indicated by propidium iodide. Scale bar 5 μM.

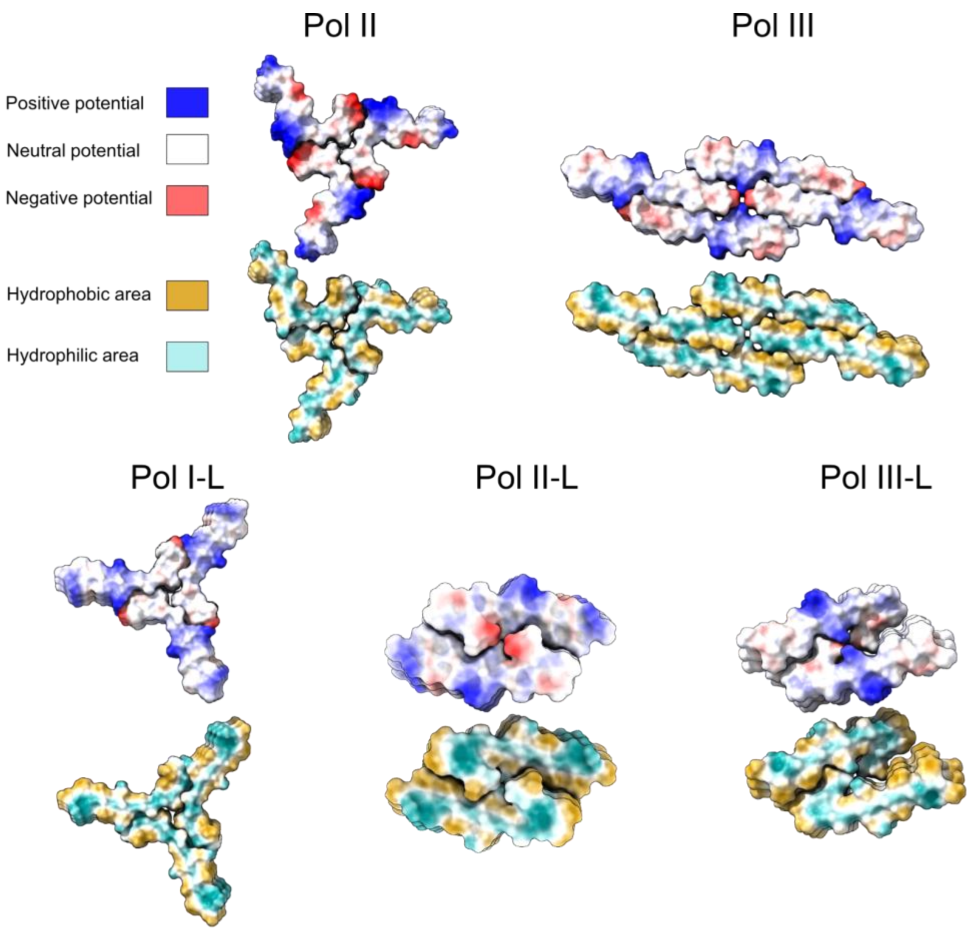

Figure S19. Electrostatic and hydrophobic surfaces of citropin 1.3 polymorphs. Surface representation of different citropin 1.3 polymorphs colored by calculated electrostatic and hydrophobic surface. Color codes of surface properties are shown.
